# Use Your Words Carefully: What Is a Chronic Disease?

**DOI:** 10.3389/fpubh.2016.00159

**Published:** 2016-08-02

**Authors:** Stephanie Bernell, Steven W. Howard

**Affiliations:** ^1^Oregon State University, Corvallis, OR, USA; ^2^Saint Louis University, Saint Louis, MO, USA

**Keywords:** chronic disease, chronic illness, HIV, health policy, health knowledge, chronic conditions

## Overview

One important element of effective communication is having a shared language or at least a shared understanding of the meaning of the central words used in a conversation. One term that is often used in discussions between patients and medical providers, in the academic literature, and in policy discussions, is “chronic disease.” There is not only tremendous variation in the diseases that are included under the umbrella term “chronic disease” but also variation in the time a disease must be present for something to be referred to as chronic. Furthermore, there is a move to include chronic conditions that are not indicators of disease, but long-standing functional disabilities, including developmental disorders and visual impairment (1–4).

Within professional communities (i.e., medical, public health, academic, and policy), there is a large degree of variation in the use of the term chronic disease. For example, the Centers for Disease Control (CDC) classify the following as chronic diseases: heart disease, stroke, cancer, type 2 diabetes, obesity, and arthritis (5). The Centers for Medicare and Medicaid Services have a more extensive list of 19 chronic conditions that includes Alzheimer’s disease, depression, and HIV, to name a few. This difference, within the Department of Health and Human Services alone, although not surprising to those in the field, has the potential to create confusion and misunderstanding when speaking in generalities about the impact of chronic disease, the cost of chronic disease, and overall measures to reduce chronic disease.

The academic literature is not immune to the same kind of terminology variation. Differences in how “chronic disease” is used are largely dependent on the data used for the research and the discipline of the lead authors (i.e., public health and sociology). For example, one study, authored by individuals from Harvard Medical School, explored the prevalence of chronic disease using NHANES data (1999–2004). The study classifies the following as chronic diseases: cardiovascular disease, hypertension, diabetes mellitus, hypercholesterolemia, asthma, COPD, and previous cancer (6). Another academic study on chronic disease, authored by a geriatrician, classifies chronic illness as “conditions that last a year or more and require ongoing medical attention and/or limit activities of daily living” (7). The implication of a non-uniform use of the term is that a detailed read of each study is necessary to avoid erroneous conclusions regarding interventions necessary to reduce chronic disease burden for the individual and society.

Popular Internet sources used by the general public to gather medical information use the terms “chronic disease” or “chronic condition” to mean slightly different things. For example, MedicineNet describes a chronic disease as,

one lasting 3 months or more, by the definition of the U.S. National Center for Health Statistics. Chronic diseases generally cannot be prevented by vaccines or cured by medication, nor do they just disappear (8).

According to Wikipedia a chronic condition is,

a human health condition or disease that is persistent or otherwise long-lasting in its effects or a disease that comes with time. The term *chronic* is often applied when the course of the disease lasts for more than three months. Common chronic diseases include arthritis, asthma, cancer, COPD, diabetes and viral diseases such as hepatitis C and HIV/AIDS (9).

Finally, the World Health Organization states that chronic diseases,

are not passed from person to person. They are of long duration and generally slow progression. The four main types … are cardiovascular diseases (like heart attacks and stroke), cancers, chronic respiratory diseases (such as chronic obstructed pulmonary disease and asthma) and diabetes (10).

The CDC’s Chronic Disease Overview omits chronic respiratory conditions, such as COPD and asthma, and makes no mention of duration of the disease or symptoms. MedicineNet’s definition does not list specific diseases, but does include the phrase “cannot be cured by medication.” Similar to MedicineNet, Wikipedia uses the 3-month time span as a marker, but does list specific diseases, including HIV. The WHO’s definition would eliminate HIV as a chronic disease as the virus is “passed from person to person.”

The variation in meaning is amplified when viewed in an international context. For example, the Australian Institute for Health and Welfare includes the following as common features of chronic disease (11):

- complex causality, with multiple factors leading to their onset
- a long development period, for which there may be no symptoms
- a prolonged course of illness, perhaps leading to other health complications
- associated functional impairment or disability.

Highlighted prominently in the information from the Australian government on chronic disease is the disease burden of mental illness and oral disease. Both of these conditions are often excluded from the chronic disease conversation in the United States (12, 13).

Given the worldwide dissemination of medical information, the variation in public information is not only confusing on paper but also has real implications for those managing chronic diseases or conditions. It is possible that recommendations for chronic disease management are missed by individuals who do not know that the information applies to them; conversely, individuals may use the recommendation when it is not advisable to do so. For example, the CDC lists “cancer” as a chronic disease when, in fact, only certain types of cancers (i.e., multiple myeloma) can be viewed in terms of a chronic illness. Other types of cancers have little treatment options and prove fatal in the near term.

## Diseases Can Transition from Fatal to Chronic

To the public health and medical community, transitions in disease states – from terminal diagnosis to chronic disease, or from acute to chronic – are not unexpected. For example, approximately 1.2 million people in the United States are living with HIV, with 50,000 new cases confirmed each year (14). Today, people with HIV are most often treated with once-a-day, fixed-dose pills, taken for the rest of his or her life. It is a vast improvement from early HIV treatment that involved a highly complex pill regimen, with difficult to manage side effects. The advances in HIV treatment have changed the life trajectory for a newly diagnosed HIV-positive individual. As of 2015, the lifespan of a person living with HIV was about the same as an individual not diagnosed with HIV (15–19).

However, a search of news articles from two national news sources (New York Times and Washington Post) from 1/1/2015 to 5/1/2016 generated zero news articles containing the words “HIV, Chronic and Disease/Condition.” If the general public is relying on these types of news sources to understand the changing nature of chronic disease, it is understandable that HIV is not typically thought of in the same category as diabetes or COPD, and the stigma of HIV as a “death sentence” remains. It is reasonable to assume that the general public is unaware that HIV-positive individuals who have a greater life expectancy than someone diagnosed with diabetes.

With the advances in HIV treatment, HIV is now a risk factor for other chronic diseases, such as cardiovascular diseases and diabetes. Patients, clinicians, public health professionals, and others interested in reducing the public health and economic burdens of chronic disease may benefit from viewing HIV not as a single chronic disease, but as a precursor to other chronic diseases (20–22).

## Looking to the Future

The National Health Council reports that the United States bears a cumulative annual economic burden of $1.3 trillion from the seven most prevalent chronic conditions – cancer, diabetes, hypertension, stroke, heart disease, pulmonary conditions, and mental illness (23). This number does not include a whole host of other chronic conditions and diseases, such as HIV. If we want to reduce the health effects and fiscal burden of chronic disease, the conversation needs to change. Of course, we need to promote lifestyle changes and medical breakthroughs to reduce chronic disease, but we also need patients, providers, policymakers, and those promoting public discourse, to be precise in the words we use to describe health, disease, and illness.

Rather than adhering to a specific list of diseases and a specified time period, we advocate for a simpler approach. According to Merriam Webster, “chronic” is something that is “continuing or occurring again and again for a long time.” Using this simpler view, we would exclude something like a broken leg as a chronic condition, but would include reoccurring lower back pain, or hormone-related migraine headaches, for example. Diseases, conditions, and syndromes that do not make the top seven list, but when taken together affect a large number of individuals who can be quite costly to manage and are justifiably emotionally and physically taxing for patients and their caregivers. By reframing the conversation, we are not advocating for drawing attention away from heart disease, diabetes, arthritis, and COPD – the most commonly discussed chronic diseases – but we are in favor of bringing more diseases (and conditions) under the umbrella, with the hope of increasing awareness, sharing knowledge, and creating a larger community of individuals working toward improving the health of those who suffer from chronic health problems.

## Author Contributions

SB: conceptualized paper topic, was the lead author of the manuscript, and finalized information for submission. SH: participated in the writing of the paper, provided a meticulous editing of the paper, and reviewing for overall impact.

## Conflict of Interest Statement

The authors declare that the research was conducted in the absence of any commercial or financial relationships that could be construed as a potential conflict of interest.
